# Crown–Root Ratio as a Predictive Morphometric Indicator in Mandibular First Molars with Occlusal Trauma and Periodontitis: A Radiographic and Clinical Modeling Study

**DOI:** 10.3390/dj13090419

**Published:** 2025-09-12

**Authors:** Lidya Irani Nainggolan, Bramma Kiswanjaya, Menik Priaminiarti, Sri Lelyati Chaidar Masulili, Hanna H. Bachtiar-Iskandar, Yuniarti Soeroso, Pitu Wulandari, Eha Renwi Astuti, Akihiro Yoshihara

**Affiliations:** 1 Doctoral Program, Faculty of Dentistry, Universitas Indonesia, Jl Salemba Raya No. 4, Central Jakarta 10430, Indonesia; lidya.irani.ngl@gmail.com; 2 Department of Dentomaxillofacial Radiology, Faculty of Dentistry, Universitas Indonesia, Jl Salemba Raya No. 4, Central Jakarta 10430, Indonesia; menik-pad@ui.ac.id (M.P.); hanna@ui.ac.id (H.H.B.-I.); 3 Department of Periodontology, Faculty of Dentistry, Universitas Indonesia, Jl Salemba Raya No. 4, Central Jakarta 10430, Indonesia; lelyati@ui.ac.id (S.L.C.M.); yuniarti@ui.ac.id (Y.S.); 4 Department of Periodontology, Faculty of Dentistry, Universitas Sumatera Utara, Jl. Alumni No. 2, Medan 20155, Indonesia; pitu.wulandari@usu.ac.id; 5 Department of Oral and Maxillofacial Radiology, Faculty of Dentistry, Universitas Airlangga, Jl. Mayjen Prof. Dr. Moestopo 47, Surabaya 60132, Indonesia; eha-r-a@fkg.unair.ac.id; 6 Division of Oral Science for Health Promotion, Department of Oral Health Science and Promotion, Graduate School of Medical and Dental Sciences, Niigata University, Niigata City 951-8514, Japan; akihiro@dent.niigata-u.ac.jp

**Keywords:** crown–root ratio, trauma from occlusion, mandibular molar, root morphology, periapical radiograph

## Abstract

**Background/Objectives:** Trauma from occlusion (TFO) is a modifying factor in periodontal disease progression, yet its morphometric impact on mandibular molars remains underexplored. The crown–root ratio (CRR), traditionally used in prosthodontic prognosis, may also serve as a diagnostic marker for structural changes in periodontally compromised teeth. This study evaluated the relationship between crown and root dimensions and clinical/radiographic parameters in mandibular first molars with TFO and developed predictive models emphasizing the role of CRR. **Methods:** This retrospective cross-sectional study included 99 periodontitis patients clinically and radiographically diagnosed with TFO. Digitized periapical radiographs of mandibular first molars (tooth 36 or 46) were analyzed to measure clinical and radiographic CRR, crown and root length, tooth inclination, alveolar bone loss, and root morphology. Correlation and stepwise multiple regression analyses identified predictors of crown and root length. **Results:** Males had significantly greater crown length (7.6 vs. 7.2 mm), root length (13.3 vs. 12.3 mm), and radiographic CRR (1.2 vs. 1.0) (*p* = 0.008). Clinical CRR showed a moderate positive correlation with crown length (*r* = 0.526) and a strong inverse correlation with root length (*r* = −0.735) (*p* < 0.001). Regression models revealed that clinical CRR, root length, and sex significantly predicted crown length (R^2^ = 0.955), while CRR and crown length predicted root length (R^2^ = 0.958). **Conclusions:** This study demonstrated that the clinical crown–root ratio (CRR) is a strong predictor of both crown and root lengths in mandibular first molars affected by trauma from occlusion (TFO) in periodontitis patients.

## 1. Introduction

Trauma from occlusion (TFO) occurs when occlusal forces exceed the adaptive capacity of the periodontium, potentially resulting in clinical and radiographic changes, such as tooth mobility, vertical bone loss, widened periodontal ligament space, and root surface alterations [1,2,3]. Although TFO has long been considered a modifying factor in periodontal disease progression, its specific impact on dental morphometry—particularly crown and root dimensions—remains insufficiently understood, especially in posterior teeth that endure the greatest masticatory forces [4,5].

One key metric that has traditionally been used to evaluate periodontal prognosis is the crown–root ratio (CRR), measured clinically or radiographically. CRR is commonly applied in prosthodontics to determine the feasibility of restorative treatments, especially in periodontally compromised teeth [6]. More recently, CRR has gained attention as a potential morphometric indicator that reflects not only anatomical proportions but also the functional impact of periodontal and occlusal loading [7]. However, the current literature predominantly focuses on anterior and premolar teeth [8,9] or uses generalized dental samples without isolating the teeth most affected by functional trauma. Limited research has specifically addressed mandibular first molars—teeth that erupt early, sustain heavy occlusal loads, and are frequently affected by bone loss in TFO cases [10].

Furthermore, although CRR has been studied descriptively, its role as a predictive variable for crown and root length and its association with clinical and radiographic parameters in the context of TFO have not been clearly quantified. In addition, most studies rely on either linear correlation or survival-based outcomes without incorporating multivariate modeling that can comprehensively assess the anatomical interplay between crown, root, and surrounding support structures. This study addresses these gaps by investigating the relationship between crown and root dimensions and various clinical and radiographic characteristics in mandibular first molars (tooth 36 or 46) of patients with periodontitis and TFO. By integrating morphometric measurements with occlusal and periodontal parameters, we aimed to develop predictive models for crown and root length, with a particular focus on the role of CRR. To our knowledge, this is the first study to model crown and root dimensions in posterior teeth affected by TFO using both clinical and radiographic CRR alongside other anatomical predictors—providing new insight into morphometric risk patterns under occlusal stress.

## 2. Materials and Methods

### 2.1. Study Design and Setting

This retrospective, cross-sectional analytic study was conducted between April and December 2023 at the Dental Hospital and Radiology Department of the Faculty of Dentistry, Universitas Indonesia. Ethical approval was obtained from the institution’s Research Ethics Committee (No. 13/Ethical Approval FKGUI/IV/2023; Protocol No. 070220323; Approval date: 10 April 2023). All procedures complied with the principles outlined in the Declaration of Helsinki, and patient data were anonymized to maintain confidentiality.

### 2.2. Study Population and Sampling

The study analyzed digitized periapical radiographs of mandibular first molars (tooth 36 or 46) from patients diagnosed with periodontitis and treated between January 2020 and December 2023. Eligible cases demonstrated both clinical and radiographic evidence of trauma from occlusion (TFO). The diagnosis of periodontitis was established according to the 2017 World Workshop Classification of Periodontal and Peri-Implant Diseases and Conditions [11], based on both clinical and radiographic parameters. Inclusion criteria included a confirmed diagnosis of periodontitis supported by comprehensive clinical and radiographic assessments. Radiographs had to be of sufficient diagnostic quality, with full visualization of the target tooth and adjacent structures. Clinical indicators of TFO included pathologic tooth mobility, premature occlusal contact, and deep infrabony periodontal pockets. Radiographic confirmation of TFO was based on the presence of vertical bone loss, a widening of the periodontal ligament space, and thinning or loss of continuity of the lamina dura. TFO diagnosis was therefore based on a combination of clinical and radiographic findings, rather than radiographic features alone. Exclusion criteria included radiographs with poor image quality, including distortion, underexposure, or loss of anatomical landmarks, which could compromise measurement accuracy. Subjects were also excluded if the targeted mandibular molars exhibited severe malocclusions (e.g., scissor bite or crossbite) or had undergone extensive restorative procedures, such as full-coverage crowns, root canal treatments, or prosthetic modifications, which could alter natural tooth morphology. Additionally, patients with systemic conditions known to affect bone metabolism or periodontal health, including uncontrolled diabetes, osteoporosis, or immunosuppressive disorders, were excluded. A history of orthodontic treatment involving the mandibular posterior region was also considered a basis for exclusion due to its potential influence on tooth positioning and morphology.

The minimum sample size was calculated using G*Power 3.1 software for multiple linear regression with five predictors, a power of 0.95, an alpha of 0.05, and an effect size of 0.3. This yielded a minimum of 70 samples. A nonrandom consecutive sampling method was employed to minimize selection bias while ensuring adequate representation. A total of 99 periodontitis patients clinically and radiographically diagnosed with trauma from occlusion (TFO) were included in this study, comprising 68 females and 31 males.

### 2.3. Operational Definitions and Measurements

**Figure 1:**
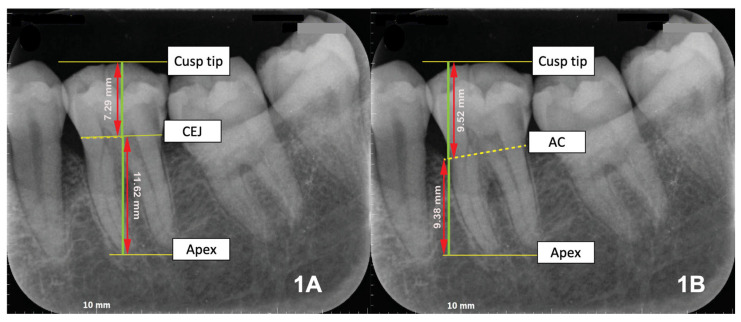
Clinical and Radiographic Crown–Root Ratios (CRR). (**A**) Clinical CRR: ratio of crown length (cusp tip–cemento-enamel junction [CEJ]) to root length (CEJ–apex), representing anatomical proportion. (**B**) Radiographic CRR: ratio of crown height (cusp tip–alveolar crest [AC]) to root length (AC–apex), reflecting remaining periodontal support. CEJ = cemento-enamel junction; AC = alveolar crest.

All intraoral periapical radiographs were acquired using the right-angle technique with a standardized X-ray system (CS 2200, Carestream Dental, Atlanta, GA, USA), operating at 70 kVp and 7 mA with exposure times ranging from 0.1 to 0.32 s, depending on patient characteristics. Although variations in patient positioning are inherent in retrospective datasets, only radiographs with clear anatomical landmarks and adequate diagnostic quality were included to minimize measurement bias. All films were analog and subsequently digitized at ≥600 dpi using a high-resolution scanner for analysis. Morphometric evaluations were conducted on digitized periapical radiographs using Adobe Illustrator (SPS), with standardized calibration and consistent contrast and magnification settings to enhance anatomical visibility. The anatomical landmarks and variables assessed in this study included both categorical and continuous morphometric parameters derived from digitized periapical radiographs, as follows:

Radiographic parameters were assessed on digitized periapical images and included the clinical crown–root ratio (CRR), calculated according to Lind (1972) [12] as the ratio of crown length (cusp tip–cemento-enamel junction [CEJ]) to root length (CEJ–apex) (Figure 1A), and the radiographic CRR, defined as the ratio of crown height (cusp tip–alveolar crest) to root length (alveolar crest–apex) (Figure 1B).

**Figure 2:**
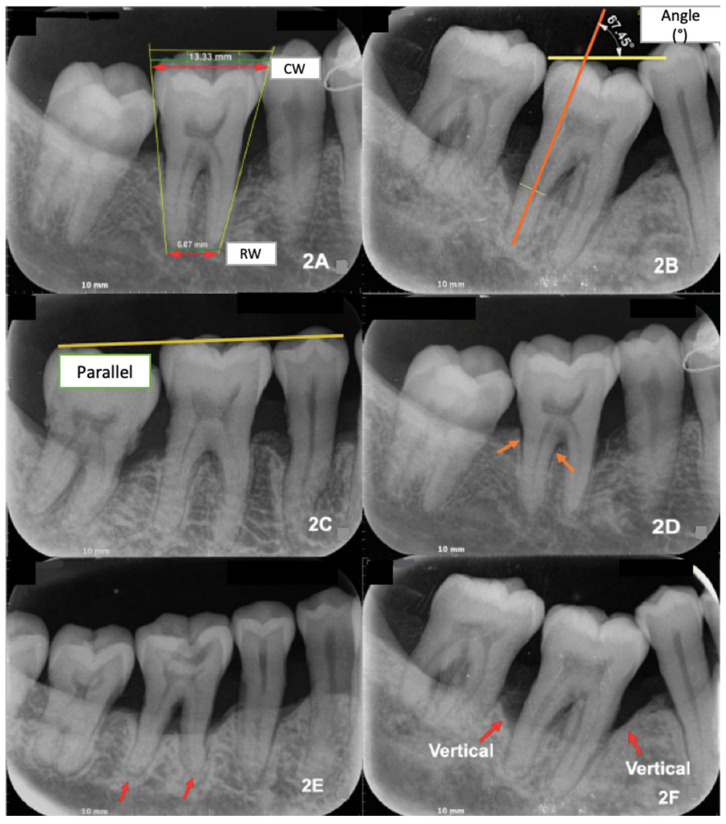
Radiographic parameters evaluated in mandibular first molars. (**A**) Crown width (CW) and root width (RW) with root convergence (occlusal vs. apical third). (**B**) Tooth axis inclination relative to occlusal plane (°). (**C**) Occlusal arch alignment (parallel vs. non-parallel) (**D**) Periodontal ligament (PDL) space widening and lamina dura thinning. (**E**) Root morphology (normal, blunted, or curved roots). (**F**) Pattern of alveolar bone loss (horizontal vs. vertical).

Additional parameters comprised crown and root width (with root convergence derived from occlusal–apical width differences) (Figure 2A), tooth axis inclination relative to the occlusal plane (Figure 2B), occlusal arch alignment (parallel vs. non-parallel) (Figure 2C), periodontal ligament space widening and lamina dura changes (absent vs. present) (Figure 2D), root morphology (normal, blunted/curved on one root, or both roots) (Figure 2E), and pattern of alveolar bone loss (horizontal, vertical on one surface, or vertical on both surfaces) (Figure 2F). Clinical parameters, including tooth mobility (Miller’s classification), premature occlusal contact (articulating paper/shimstock), probing pocket depth, and infrabony defects, were obtained from patient records.

All measurements were performed by a calibrated examiner. Reliability was assessed using Cohen’s kappa for categorical variables and intraclass correlation coefficients (ICC) for continuous variables, based on repeated evaluations of 50% of the sample.

### 2.4. Data Collection and Analysis

Radiographs meeting the inclusion criteria were digitized and evaluated morphometrically. Clinical data were concurrently extracted from corresponding medical records. All measurements were conducted in a standardized manner by a single examiner to minimize inter-observer variability, with calibration repeated during the process to ensure consistency.

Statistical analysis was performed using IBM SPSS Statistics version 22.0. Descriptive statistics summarized the distribution of clinical and radiographic parameters. For bivariate analysis, chi-square tests were applied to categorical variables, and either independent samples *t*-tests or independent samples median tests were used for numerical variables, depending on data normality. Multivariate analysis was carried out using stepwise multiple linear regression to identify independent predictors of crown and root length. Model performance was evaluated using the coefficient of determination (*R^2^*) and diagnostic indices.

## 3. Results

The reliability analysis demonstrated high agreement for both the continuous and categorical measurements. The intraclass correlation coefficient (ICC) for continuous variables of clinical CRR was 0.996 for intra-observer and 0.993 for inter-observer assessments, indicating excellent agreement. For categorical parameters, Cohen’s kappa values for root morphology were 0.932 (intra-observer) and 0.856 (inter-observer), both reflecting a high level of agreement.

As shown in Table 1, the median age was 46 years for females and 42 years for males, with no statistically significant difference between the sexes. Clinical parameters, including tooth mobility, premature occlusal contact, and probing pocket depth (PPD), showed comparable distributions across the sexes, with infrabony pockets being predominant. Radiographically, unilateral periodontal space widening, and lamina dura thinning were observed in 65.7% of the females and 31.3% of the males. Vertical bone loss patterns—particularly involving both mesial and distal surfaces—were more frequent than horizontal defects, and nonparallel occlusal arch alignment was more common than parallel alignment. No subjects exhibited completely normal root morphology; most presented with blunt or curved roots on both the mesial and distal aspects. Among morphometric variables, the males had significantly greater crown length (7.6 mm vs. 7.2 mm; *p* = 0.025), root length (13.3 mm vs. 12.3 mm; *p* = 0.008), and radiographic crown–root ratio (1.2 vs. 1.0; *p* = 0.008) compared with the females. Other parameters, including clinical CRR, crown width, root width, tooth axis inclination, and interproximal bone loss, showed no statistically significant sex-related differences.

Table 2 presents the results of Spearman correlation analysis. Crown length was moderately correlated with clinical CRR (*r* = 0.526, *p* < 0.001) and weakly, but significantly, correlated with radiographic CRR (*r* = 0.236, *p* = 0.019) and crown width (*r* = 0.291, *p* = 0.003). A weak correlation with sex was also observed (*r* = 0.237, *p* = 0.018), with females showing longer crowns. Root length showed a strong inverse correlation with clinical CRR (*r* = −0.735, *p* < 0.001) and moderate positive correlations with radiographic CRR (*r* = 0.366, *p* < 0.001), root width (*r* = 0.308, *p* = 0.002), and sex (*r* = 0.312, *p* = 0.002), indicating that males tended to have longer roots. Most categorical clinical and radiographic variables were not significantly correlated with crown or root length, except for the pattern of alveolar bone loss, which was significantly associated with root length (*r* = −0.228, *p* = 0.023).

Table 3 shows the stepwise multiple linear regression analysis for crown length. In Model 1, clinical CRR alone was a strong predictor (*β* = 0.632, *p* < 0.001; *R^2^* = 0.400). The addition of root length in Model 2 substantially improved explanatory power (*R^2^* = 0.945), with both variables remaining highly significant. Subsequent models revealed small but significant contributions from sex (*β* = 0.068, *p* = 0.005), root morphology (*β* = −0.057, *p* = 0.013), and age (*β* = −0.046, *p* = 0.038), with the final model explaining 95.5% of the variance in crown length.

Table 4 presents the regression analysis for root length. CRR was inversely associated with root length in Model 1 (*β* = −0.703, *p* < 0.001; *R^2^* = 0.494). The addition of crown length in Model 2 dramatically increased the model’s explanatory capacity (*R^2^* = 0.954). Root morphology was added in Model 3 (*β* = 0.050, *p* = 0.022), followed by sex in Model 4 (*β* = −0.045, *p* = 0.045), yielding a final *R^2^* of 0.958. Together, these findings highlight the dominant roles of clinical CRR and anatomical crown and root dimensions in predicting one another, while demographic and morphological features provided smaller but significant contributions. Collinearity diagnostics indicated that all predictors had VIF values below 2.1, confirming the absence of multicollinearity and supporting the validity of the regression models (Table 3 and Table 4).

## 4. Discussion

This study provides a comprehensive clinical and radiographic assessment of mandibular first molars in periodontitis patients with trauma from occlusion (TFO). By analyzing a single posterior tooth type, we reduced anatomical variability and emphasized the morphometric changes associated with both periodontal destruction and occlusal overload. Mandibular first molars were specifically chosen because of their early eruption, heavy functional loading, and frequent involvement in occlusal trauma; however, this focus limits the generalizability of our findings to other tooth types and regions. In the broader prognostic context, CRR should therefore be interpreted as a supplementary morphometric marker that complements established periodontal and prosthodontic prognosis frameworks [5,6]. 

One of the most significant findings of this study is the strong predictive role of the clinical crown–root ratio (CRR) for both crown and root length. The observed moderate-to-strong positive correlation between CRR and crown length (*r* = 0.526, *p* < 0.001), as well as the strong inverse correlation between CRR and root length (*r* = −0.735, *p* < 0.001), confirms that CRR is closely associated with morphometric variation in mandibular first molars. Our regression models further reinforce this interpretation, with CRR and root length explaining up to 95.5% of the variance in crown length, while CRR and crown length explained 95.8% of the variance in root length. These findings demonstrate a tightly linked anatomical relationship, while demographic variables such as sex and age, as well as root morphology, had comparatively minor effects. The collinearity statistics further supported the robustness of these models, as all predictors demonstrated VIF values below 2.1, indicating no multicollinearity among the independent variables.

These findings are consistent with prior studies reporting the prognostic and morphometric significance of CRR. Shi et al. (2024) found that unfavorable CRR values were strongly associated with reduced tooth survival in patients with advanced periodontitis [10]. Yun et al. (2014) provided population-based reference values for CRR in healthy permanent teeth, demonstrating natural variation according to tooth type and sex [13]. Tang et al. (2024), using CBCT analysis, showed that crown–root morphology is also associated with alveolar bone characteristics in anterior teeth [14]. Compared with these studies, our investigation is unique in focusing specifically on mandibular first molars subjected to occlusal trauma, thereby extending the application of CRR as a morphometric indicator to posterior teeth under high functional loading [13,14,15].

Potential clinical implications should be interpreted cautiously. Although not directly tested in this study, the strong statistical association between CRR and morphometric parameters suggests that CRR may serve as a functional and diagnostic surrogate for periodontal and morphological changes under occlusal stress [3,4,5]. Beyond its traditional role in prosthodontic prognosis, possible applications of CRR include serving as a supplementary marker for periodontal prognosis, informing decisions on tooth preservation versus extraction, and guiding the selection of abutment teeth for prosthetic rehabilitation [6,16]. In periodontal therapy, unfavorable CRR values may indicate reduced regenerative potential, necessitating cautious long-term maintenance. In orthodontics, variations in root length are relevant for assessing the risk of root resorption under mechanical loading [17,18]. In occlusal rehabilitation, CRR assessment could help anticipate biomechanical challenges and guide preventive or interceptive interventions. Collectively, these considerations underscore the potential clinical utility of CRR across multiple dental disciplines, though longitudinal and validation studies remain essential to confirm its prognostic relevance.

Several limitations of this study should be acknowledged. First, the cross-sectional design restricts causal inference; therefore, longitudinal studies are needed to confirm the directionality of the observed relationships. In addition, although the diagnosis of periodontitis was standardized using the 2017 World Workshop Classification, subgroup analyses according to staging or mobility cut-off values were not feasible due to the retrospective design and limited sample distribution. Future studies with larger and prospectively collected datasets are needed to address these subgroup differences. Second, the focus on mandibular first molars improved internal validity but may limit generalizability to other tooth types; future studies should include additional tooth regions and, ideally, a comparison group of periodontally healthy molars to further validate the diagnostic utility of CRR. Third, the use of two-dimensional radiographs limited the assessment of root morphology, and the absence of quantitative occlusal force measurements may have reduced the precision of TFO evaluation. Future research using three-dimensional imaging modalities (e.g., CBCT [19]) in combination with digital occlusal analysis tools, such as T-Scan or three-dimensional force measurement systems [20], could provide stronger and more quantitative biomechanical correlations. Fourth, although the regression models demonstrated excellent fit, the very high proportion of variance explained (>95%) raises the possibility of model overfitting, particularly given the modest sample size. External validation with independent datasets or prospective cohorts is recommended to confirm the stability and generalizability of these predictive models. Despite these limitations, the study contributes novel evidence on the role of CRR as a morphometric indicator in periodontally compromised mandibular molars under occlusal trauma.

## 5. Conclusions

This study demonstrated that the clinical crown–root ratio (CRR) is a strong predictor of both crown and root lengths in mandibular first molars affected by trauma from occlusion (TFO) in periodontitis patients. The multivariate regression models explained over 95% of the variance in these morphometric dimensions, highlighting CRR as a robust indicator of tooth morphology under occlusal stress. These findings underscore the potential clinical utility of CRR not only as a traditional prognostic tool but also as a morphometric marker for assessing structural adaptations of periodontally compromised posterior teeth. Nevertheless, the reliance on two-dimensional radiographs represents an inherent limitation of this study, and future research should integrate three-dimensional imaging modalities, such as cone-beam computed tomography (CBCT), to achieve a more precise evaluation of root morphology and to validate the associations observed.

## Figures and Tables

**Table 1:** Descriptive characteristics of subjects by sex.

Variables	Sex		*p*-Value
	Female	Male	
Age (years) *	46 (20–70)	42 (30–77)	Not significant (independent samples median test)
**Clinical Examination**			
Tooth mobility 0: No	50 (50.5%)	23 (23.2%)	Not significant (Pearson chi-square)
1: Yes	18 (18.2%)	8 (8.1%)	
Presence of premature contact 0: No	40 (40.4%)	19 (19.2%)	Not significant (Pearson chi-square)
1: Yes	28 (28.3%)	12 (12.1%)	
Probing pocket depth (PPD) 0: Suprabony pocket	7 (7.1%)	6 (6.1%)	Not significant (Pearson chi-square)
1: Infrabony pocket	61 (61.6%)	25 (25.3%)	
**Dental radiology examination**			
Periodontal space widening and lamina dura thinning (unilateral) 0: No	3 (3%)	0 (0%)	Not significant (Fisher’s exact test)
1: Yes	65 (65.7%)	31 (31.3%)	
Pattern of alveolar bone loss 0: Horizontal loss on both mesial and distal surfaces	7 (7.1%)	6 (6.1%)	Not significant (Pearson chi-square)
1: Vertical loss on either mesial or distal surface	28 (28.3%)	16 (16.2%)	
2: Vertical loss on both mesial and distal surfaces	33 (33.3%)	9 (9.1%)	
Occlusal arch alignment 0: Parallel occlusal arch	15 (15.2%)	9 (9.1%)	Not significant (Pearson chi-square)
1: Non-parallel occlusal arch	53 (53.5%)	22 (22.2%)	
Root morphology 0: normal root shape on both mesial and distal roots	0 (0%)	0 (0%)	Not significant (Fisher’s exact test)
1: Blunt/curved root on either mesial or distal root	13 (13.1%)	7 (7.1%)	
2: Blunt/curved root on both mesial and distal roots	55 (55.6%)	24 (24.2%)	
Crown length (mm) *	7.2 (5.4–11.5)	7.6 (6.3–9.2)	*p* = 0.025 (independent samples median test)
Root length (mm) *	12.3 (9–20.4)	13.3 (11.5–16.6)	*p* = 0.008 (independent samples median test)
Clinical crown-root ratio (CRR) **	0.59 (0.11)	0.57 (0.08)	Not significant (independent samples *t*-test)
Crown width (mm) *	12.3 (2.3–14.2)	12.6 (10.4–14.6)	Not significant (independent samples median test)
Root width (mm) **	8.6 (1.4)	8.8 (1.1)	Not significant (independent samples *t*-test)
Tooth axis inclination to the occlusal arch (°) *	76.2 (55.8–86.8)	75.7 (48–86.4)	Not significant (independent samples median test)
Interproximal bone loss (mm) *	2.3 (0.5–9)	2.9 (0.5–7.8)	Not significant (Independent samples median test)
Radiographic crown-root ratio *	1 (0.8–1.9)	1.2 (0.8–1.6)	*p* = 0.008 (Independent samples median test)

* Median (minimum–maximum); ** Mean (standard deviation).

**Table 2:** Spearman correlation between crown and root length and clinical and radiographic variables.

Variables	Crown Length		Root Length	
	Correlation Coefficient (r)	*p*-Value	Correlation Coefficient (r)	*p*-Value
Age (years)	−0.171	0.09	0.035	0.729
Sex 1: Female	0.237 *	0.018	0.312 **	0.002
2: Male				
**Clinical examination**				
Tooth mobility 0: No	0.027	0.791	−0.068	0.502
1: Yes				
Presence of premature contact 0: No	0.053	0.605	0.003	0.980
1: Yes				
Probing pocket depth (PPD) 0: Suprabony pocket	0.035	0.730	−0.136	0.179
1: Infrabony pocket				
**Dental radiology examination**				
Periodontal space widening and lamina dura thinning (unilateral) 0: No	−0.134	0.186	0.056	0.584
1: Yes				
Pattern of alveolar bone loss 0: Horizontal loss on both mesial and distal surfaces	−0.099	0.331	−0.228 *	0.023
1: Vertical loss on either mesial or distal surface				
2: Vertical loss on both mesial and distal surfaces				
Occlusal arch alignment 0: Parallel occlusal arch	0.036	0.721	−0.134	0.187
1: Non-parallel occlusal arch				
Root morphology 0: normal root shape on both mesial and distal roots	−0.019	0.852	−0.129	0.203
1: Blunt/curved root on either mesial or distal root				
2: Blunt/curved root on both mesial and distal roots				
Crown length (mm)			0.142	0.16
Root length (mm)	0.142	0.16		
Clinical crown-root ratio (CRR)	0.526 **	0.000	−0.735 **	0.000
Crown width (mm)	0.291 **	0.003	0.027	0.791
Root width (mm)	0.063	0.538	0.308 **	0.002
Tooth axis inclination (°)	0.148	0.144	0.126	0.213
Interproximal bone loss (mm)	−0.134	0.186	−0.053	0.605
Radiographic crown-root ratio	0.236 *	0.019	0.366 **	0.000

*p*  <  0.05 (*), *p*  <  0.01 (**); correlation calculated using Spearman’s rank correlation test.

**Table 3:** Stepwise multiple linear regression analysis of crown length (mm) as the dependent variable.

Model		Coefficients (B)	Standard Error (SE)	Standardized Coefficients (β)	*p*-Value	Collinearity VIF
1	(Constant)	4.201	0.412		0.000	
	Clinical crown–root ratio (CRR)	5.547	0.690	0.632	0.000	1.000
	R^2^				0.400	
2	(Constant)	−6.199	0.358		0.000	
	Clinical crown–root ratio (CRR)	11.959	0.295	1.363	0.000	1.978
	Root length (mm)	0.513	0.017	1.039	0.000	1.978
	R^2^				0.945	
3	(Constant)	−6.179	0.346		0.000	
	Clinical crown–root ratio (CRR)	11.868	0.286	1.352	0.000	2.003
	Root length (mm)	0.502	0.016	1.017	0.000	2.093
	Sex (1 = Female; 2 = Male)	0.134	0.047	0.068	0.005	1.065
	R^2^				0.950	
4	(Constant)	−5.950	0.348		0.000	
	Clinical crown–root ratio (CRR)	11.899	0.278	1.356	0.000	2.007
	Root length (mm)	0.501	0.016	1.015	0.000	2.094
	Sex (1 = Female, 2 = Male)	0.131	0.046	0.066	0.005	1.066
	Root morphology ^1^	−0.130	0.051	−0.057	0.013	1.009
	R^2^				0.953	
5	(Constant)	−5.726	0.358		0.000	
	Clinical crown–root ratio (CRR)	11.850	0.274	1.350	0.000	2.021
	Root length (mm)	0.500	0.016	1.013	0.000	2.096
	Sex (1 = Female, 2 = Male)	0.132	0.045	0.067	0.004	1.066
	Root morphology ^1^	−0.132	0.050	−0.058	0.010	1.009
	Age (years)	−0.004	0.002	−0.046	0.038	1.010
	R^2^				0.955	

R^2^ = Coefficient of Determination. ^1^ Root morphology: 0 = normal root shape on both mesial and distal roots; 1 = blunt/curved root on either mesial or distal root; 2 = blunt/curved root on both mesial and distal roots. ^2^ VIF = Variance inflation Factor.

**Table 4:** Stepwise multiple linear regression analysis of root length (mm) as the dependent variable.

Model		Coefficients (B)	Standard Error (SE)	Standardized Coefficients (β)	*p*-Value	Collinearity VIF
1	(Constant)	20.272	0.766		0.000	
	Clinical crown–root ratio (CRR)	−12.499	1.283	0.703	0.000	1.000
	R^2^				0.494	
2	(Constant)	12.829	0.334		0.000	
	Clinical crown–root ratio (CRR)	−22.327	0.502	−1.256	0.000	1.665
	Crown length (mm)	1.772	0.057	0.875	0.000	1.665
	R^2^				0.954	
3	(Constant)	12.401	0.375		0.000	
	Clinical crown–root ratio (CRR)	−22.474	0.495	−1.264	0.000	1.693
	Crown length (mm)	1.785	0.056	0.881	0.000	1.682
	Root morphology ^1^	0.231	0.100	0.050	0.022	1.017
	R^2^				0.956	
4	(Constant)	12.525	0.374		0.000	
	Clinical crown–root ratio (CRR)	−22.736	0.504	−1.279	0.000	1.812
	Crown length (mm)	1.821	0.058	0.899	0.000	1.854
	Root morphology ^1^	0.231	0.098	0.050	0.021	1.017
	Sex (1 = Female; 2 = Male)	−0.180	0.089	−0.045	0.045	1.110
	R^2^				0.958	

R^2^ = Coefficient of Determination. ^1^ Root morphology: 0 = normal root shape on both mesial and distal roots; 1 = blunt/curved root on either mesial or distal root; 2 = blunt/curved root on both mesial and distal roots. ^2^ VIF = Variance inflation Factor.

## Data Availability

The data that support the findings of this study are available on request from the corresponding author (B.K.). The data are not publicly available due to privacy or ethical restrictions.
